# A new tropical cyclone surge index incorporating the effects of coastal geometry, bathymetry and storm information

**DOI:** 10.1038/s41598-021-95825-7

**Published:** 2021-08-18

**Authors:** Md. Rezuanul Islam, Chia-Ying Lee, Kyle T. Mandli, Hiroshi Takagi

**Affiliations:** 1 Department of Transdisciplinary Science and Engineering, School of Environment and Society, Tokyo Institute of Technology, Tokyo, 152-8550 Japan; 2 Lamont-Doherty Earth Observatory, Columbia University, Palisades, NY 10964 USA; 3 Department of Applied Physics and Applied Mathematics, Columbia University, Palisades, NY 10964 USA

**Keywords:** Natural hazards, Ocean sciences

## Abstract

This study presents a new storm surge hazard potential index (SSHPI) for estimating tropical cyclone (TC) induced peak surge levels at a coast. The SSHPI incorporates parameters that are often readily available at real-time: intensity in 10-min maximum wind speed, radius of 50-kt wind, translation speed, coastal geometry, and bathymetry information. The inclusion of translation speed and coastal geometry information lead to improvements of the SSHPI to other existing surge indices. A retrospective analysis of SSHPI using data from 1978–2019 in Japan suggests that this index captures historical events reasonably well. In particular, it explains ~ 66% of the observed variance and ~ 74% for those induced by TCs whose landfall intensity was larger than 79-kt. The performance of SSHPI is not sensitive to the type of coastal geometry (open coasts or semi-enclosed bays). Such a prediction methodology can decrease numerical computation requirements, improve public awareness of surge hazards, and may also be useful for communicating surge risk.

## Introduction

Storm surge associated with tropical cyclones has a long history of causing catastrophic damage and many deaths along low-elevation (< 10 m) coastal zones. Based on a 2003 study, storm surge may be responsible for as many as 2.6 million deaths worldwide during the past 200 years^1^. As the Earth’s climate warms because of human activities, a more severe, widespread storm surge hazard is projected with high confidence due to both the rising sea-level and the possible increase in TC intensity^2^. Furthermore, coastal development results in high population density in low-lying cities roughly five times (241 people/km^2^) than the global mean (47 people/km^2^)^3^. The storm surge threat has never been greater and such concern is exemplified by several recent extreme surge events, such as Hurricane Katrina (2005) that generated a peak surge of 8 m and made it one of the costliest ($149 billion) natural disasters in the United States (US) history^4^. More recently, an 8 m storm surge due to Typhoon Haiyan (2013) killed 6300 people and left 1061 missing in the Philippines^5^. Precise and timely forecasts informing effective warnings are imperative to mitigate the risks to life and property posed by TCs and its associated storm surges^6–8^.

When quantifying and communicating natural disasters such as TC, practitioners and scientists have often employed categorization-based statistical approaches for ease of public understanding and usefulness. For instance, the Saffir–Simpson hurricane wind scale (SSHWS) has been using for nearly five decades by the National Hurricane Center (NHC) in the US to categorize TC strength. Coastal inhabitants in the US thus have learned to assess the danger of TCs using SSHWS, e.g., evacuation intent increases linearly with SSHWS category^9^. Other TC prone countries such as Japan^10^, Bangladesh^11^, Australia^12^ also warn their coastal inhabitants employing a wind intensity-based scale, similar to SSHWS. However, these scales are defined based on wind-induced structural damage and do not account for other crucial factors that influence surge generation^13^. Thus, SSHWS has been widely criticized as an inappropriate estimate of storm surge potential^14–16^.One argument is that although TCs are often weakening during the landfall time frame, the storm surge potential may still be increasing at the same time. Thus, a lower category TC (i.e., Category 1, 2 in SSHWS) can sometimes inflict a significant storm surge. In the 2003–2008 period, hurricanes have generated three of the largest five surges occurring in the US within the past 65 years. Yet none of these hurricanes registered higher than a category 3 hurricane at landfall^15^. Hurricane Sandy (2012) highlighted the hazard posed by a weakening TC. Sandy approached the United States coast as a category 3 hurricane, before weakening and making landfall as a post-tropical storm. It generated ~ 2.7 m storm surge and resulted in more than 60 direct deaths from drowning^17^. Another recent example is TC Amphan (2020) which approached India–Bangladesh coast as a category 5 hurricane. Although, Amphan made landfall as a category 1 hurricane, it resulted in ~ 2.75 m storm surge and claimed hundreds of lives^18^. Considering storm surge is an extremely life-threatening hazard, there is an obvious need for an alternative means of more effectively characterizing TC surge potential.

Numerical simulation-based surge modeling also shows that wind speed is not the only storm parameter that markedly influences surge extent. Weisberg and Zheng^19^ found that the greatest storm surge events would occur when a hurricane makes landfall to the north of Tampa Bay (US), resulting in maximum winds at the mouth (i.e., the south) of the bay. Irish, Resio, and Ratcliff^15^ evaluated the relationship between hurricane size (radius to maximum wind speed: *R*_*max*_) and maximum storm surge over idealized continental shelf slopes. Their results demonstrated that storm surges tend to increase with hurricane size and that this relationship becomes increasingly pronounced for shallow coastal waters. Sebastian et al.^20^ found that storm surge behavior is highly sensitive to the local wind direction and landfall location. These findings support the conclusion of our recent work^21^, in which we showed that storm surge characteristics in a semi-enclosed bay, such as Tokyo bay in Japan, is largely sensitive to the landfall location, local wind direction, and storm size.

Other factors, including TC forward speed and coastal geometry are also found to be influential to surge generation in many previous studies. For example, Jelesnianski^22^ performed numerical experiments and found that a fast-moving TC (> 48.2 km/h) tended to intensify the storm surge. This tendency was also reported by Rego and Li^23^, who used Hurricane Rita (2005) as a reference storm; they demonstrated that faster propagation speed resulted in a greater surge but decreased the potential of the largest flood area along the open coasts of the Louisiana-Texas shelf. However, Peng, Xie, and Pietrafesa^24^ found that both the surge height and inundation areas over the Croatan-Albemarle-Pamlico Estuary decreased as hurricane forward speed increased. Using 42 years of tidal records and landfall TC best tracks in Japan, Islam and Takagi^25^, showed that fast-moving TCs tended to amplify the storm surge along open coastlines but reduced the surge at semi-enclosed bays (vice-versa). Several recent studies^26,27^ suggested that the forward speed of TCs has decreased significantly both at the global and regional scales. It indicates that coastal areas have experienced a longer influence of time by TCs. Therefore, it is of great interest to incorporate TC forward speed and coastal geometry information in the surge index.

Table 1 lists the existing surge indices. None of them considered TC forward speed with the exception of Van Ormondt et al.^28^ and coastal geometry (surge estimates for bays and open coasts separately). Another common limitation is that some of them can only be applied to NHC’s responsible area because they use TC structure variables (i.e., the radius of 64-kt wind (*R*_*64*_), *R*_*max*_) that are only available in the Atlantic hurricane forecasting database^29^ (NHC data archive; Table 1). It also needs to be noted that using *R*_*64*_ limits the usage of indices given that TCs do not always have 64-kt winds. Here, we present a new surge index that uses TC characteristics from best track data combined with regional bathymetry information to predict TC’s peak surge potential with varying coastal geometry. Our approach is more advanced than existing indices in that we add TC forward speed and coastal geometry; meteorological parameters used in the new index are those most common and often readily available at real-time in TC forecasted track data. As shown in the analysis of the new index section, we will apply the surge index to predict TC-induced peak surge potential in Japan. We focus on the Japanese coastlines due to its geographical uniqueness with various types of coastal geometry and the availability of an extensive long-term dataset pertaining to TC best track and tide data provided by the Japan Meteorological Agency (JMA). Our simplified approach provides an instantaneous overall estimate of a TC’s peak surge potential, which can supplement computationally expensive TC surge model forecasts. By providing a potential peak surge, the proposed index can be used to inform the public and emergency responders as a means of quantifying surge hazard effectively, similar to the role the SSHWS plays for wind hazard during a TC event.

**Table 1:** A comparison of the characteristics of existing storm surge indices.

Index	Predictand	Predictors									
		TC intensity		TC size				TC forward speed	Variation in coastal geometry (separating open coasts and bays)	Bathymetry information	Other variables
		Maximum sustained wind speed (*V*_*max*_)	Pressure deficit at the center of the TC (*∆P*)	Radius to maximum wind speed (*R*_*max*_)	Radius to 34-kt wind speed (*R*_*34*_)	Radius to 50-kt wind speed (*R*_*50*_)	Radius to 64-kt wind speed (*R*_*64*_)				
Saffir-Simpson hurricane scale^30,31^ (current name: SSHWS)	Categorized surge	×									
Hurricane surge index (Eq. 1) ^32,33^	Storm surge impact	×					×				
Surge damage potential (Eq. 2) ^14^	Surge damage potential				×		×				
Surge scale (Eq. 3) ^34^	Storm surge		×				×			×	
Tropical cyclone surge index^35^	Surge damage potential	×									Area of surface wind speed greater than 34-kt
Kuykendall scale^36^	Storm surge impact										Fiscal damages from storm surge flooding, ADCIRC simulation output data
Semi empirical storm surge prediction ^28^	Storm surge	×		×	×			×			Distance from landfall location, track angle, inflow angle, continental slope and shelf width, Delft3D-FLOW simulation output data

## Historical indices

After the devastating damages incurred by Hurricane Katrina in 2005, Kantha^32,33^ was one of the first who criticized the SSHWS and suggested a non-dimensional relationship for estimating surge damage potential (hurricane surge index, (HSI)) based on TC intensity (*V*_*max*_) and radius of hurricane force wind (*R*_*33*_):

1 $$ {\text{HSI}} = \left( {\frac{{V_{max} }}{{V_{ref} }}} \right)^{2} \left( {\frac{{R_{33} }}{{R_{ref} }}} \right) $$

*V*_*ref*_ and *R*_*re*f_ are climatological reference constants: 33 m/s and 96.6 km. This scale has the advantage of yielding a continuous scale and does not saturate at the higher end as SSHWS does at category 5. Larger values of HSI indicate more severe surge damage potential. The HSI has quadratic dependence to *V*_*max*_ because wind momentum input at the water surface is proportional to *V*^*2*^_*max*_. The reason for the linear dependence of HSI on the storm radius is because the storm surge impact is most often confined to a broad but roughly linear strip along the coastline^33^. Another underlying assumption that is not directly discussed in Kantha^32,33^ but may fit with Eq. (1) is that integration of storm size and wind strength over the footprint of the TC provides a bulk amount of energy/momentum transferred from the storm to the water column and thus the functional dependence of the total water level on the velocity and storm radius.

Following Kantha’s^32^ lead, Powell and Reinhold^14^ proposed a ranking based surge damage potential (S_DP_) for the US coasts considering the integrated kinetic energy of the hurricane wind field:

2 $$ {\text{S}}_{{{\text{DP}}}} = \, 0.{676 } + \, 0.{43}\sqrt {IKE_{TS} } - 0.0176(\sqrt {IKE_{TS} } ) - 6.5)^{2} $$

where, *IKE*_*TS*_ is the integrated kinetic energy for marine winds greater than tropical storm force (18 m/s). The larger value of S_DP_ indicates more severe surge damage potential but with an upper bound limit at 6^14^.

Later, Irish and Resio^34^ addressed the relative importance of water depth variation across the continental shelves adjacent to the US and proposed an improved dimensionless and continuous surge scale (SS) is of the form

3 $$ {\text{SS }} = \, \left( {{2}.{\text{43E }}{-}{ 4}} \right)\Delta {\text{p}}L_{30m} \Psi_{{\text{x}}} \left( {\frac{{R_{33} }}{{L_{30m} }}} \right) $$

$$\psi_{x}\,\,\left(\frac{{R}_{33}}{{L}_{30m}}\right) =\left(\frac{{R}_{33}}{{L}_{30m}}\right)\,\, {\rm when}\,\,\left(\frac{{R}_{33}}{{L}_{30m}}\right) \leq 1 \,\,{\rm and} \,\,\psi_{x}\left(\frac{{R}_{33}}{{L}_{30m}}\right)=1 \,\, {\rm when}\,\,\left(\frac{{R}_{33}}{{L}_{30m}}\right) > 1.$$

In Eq. (3), *∆p* is the hurricane central pressure difference, defined as the nominal atmospheric pressure around a hurricane minus the central pressure of that hurricane and directly proportional to the *V*^2^_*max*_. *L*_*30m*_ is the horizontal distance (km) between the shoreline and the 30 m depth contour and $(\frac{{R}_{33}}{{L}_{30m}})$ is thus the ratio of the storm size to *L*_*30m*_. *Ψ*_*x*_ is the dimensionless storm size function to adjust that ratio. *L*_*30m*_ used in the scale is because Irish and Resio^34^ found that surge generation tended to be confined between the shore and the 30-m depth contour for the five representative shelf profiles in the US. Chavas et al.^37^ has also reported similar results where they suggested *L*_*30m*_ is an optimal characteristic length scale for storm surge generation in the US coasts. Irish and Resio^34^ argued that Eq. (3) behaves fundamentally different to Eq. (1) and Eq. (2), as it introduces an upper bound of storm size by limiting *Ψ*_*x*_ $(\frac{{R}_{33}}{{L}_{30m}})$ based on $(\frac{{R}_{33}}{{L}_{30m}})$.

While SS shows improvement over the previous indices (SSHWS, HSI, and S_DP_) for estimating surge potential, it was only validated with limited observed peak open coast surge values with sample size (n) = 29, leaving no index-based method for bays or estuaries. Kantha^38^ argued that it is better to consider TC forward speed in the Eq. (3) as the TCs’ temporal response depends on the ratio of the storm residence time scale to the shelf response time scale. Again, to the best of the authors knowledge, there is no index that investigated the combined influences of TC forward speed, size, intensity, bathymetry, coastal geometry, and long-term storm surge observations over a large area (i.e., for a country or globally).

## Formulation of new index

Here we propose a dimensionless and continuous storm surge hazard potential index (SSHPI) (Eq. 4). The mathematical equation of SSHPI adopts and modifies those from Eqs. (1) and (3) (intensity ${(\frac{{V}_{max}}{{V}_{ref}})}^{2}$, size $(\frac{{R}_{50}}{{R}_{ref}})$, and bathymetry $(\frac{{L}_{30}}{{L}_{*}})$) and further adds coastal geometry parameter (*a*) and TC forward speed information (*S*) introduced in Islam and Takagi^25^:

4 $$ {\text{SSHPI }} = \left( {\frac{{V_{max} }}{{V_{ref} }}} \right)^{2} \left( {\frac{{R_{50} }}{{R_{ref} }}} \right)\left( {\frac{S}{{S_{ref} }}} \right)^{a} \left( {\frac{{L_{30} }}{{L_{*} }}} \right). $$

$$ \frac{{R_{50} }}{{R_{ref} }} = \left\{ {\begin{array}{*{20}c} {1.5} \\ {\frac{{R_{50} }}{{R_{ref} }} } \\ {0.5} \\ \end{array} \begin{array}{*{20}c} { if \frac{{R_{50} }}{{R_{ref} }} \ge 1.5 } \\ {if 0.5 < \frac{{R_{50} }}{{R_{ref} }} < 1.5} \\ {if \frac{{R_{50} }}{{R_{ref} }} \le 0.5} \\ \end{array} } \right.;\quad \left( {\frac{S}{{S_{ref} }}} \right)^{a} = \left\{ {\begin{array}{*{20}c} {1.5} \\ {\left( {\frac{S}{{S_{ref} }}} \right)^{a} } \\ {0.5} \\ \end{array} \begin{array}{*{20}c} { if \left( {\frac{S}{{S_{ref} }}} \right)^{a} { } \ge { }1.5 } \\ { if 0.5 < \left( {\frac{S}{{S_{ref} }}} \right)^{a} < 1.5 } \\ {if \left( {\frac{S}{{S_{ref} }}} \right)^{a} { } \le { }0.5} \\ \end{array} } \right. $$

Specifically, *R*_*50*_ is a measure of radius to 50-kt (26 m/s) winds (nautical mile (nm)), *S* is the forward speed (km/h), *a* is the characteristic coastal geometry^25^: for open coast, *a* = 1 and for semi-enclosed bay, *a* = − 1. As TC’s wind field can be highly asymmetrical, making it difficult to determine the actual aerial coverage of the wind field with a specific speed^29,39^, we use the arithmetic average of the longest and shortest *R*_*50*_. The reason of using *R*_*50*_ instead of *R*_*33*_ is because the former one is recorded consistently in HURDAT2 (Atlantic Hurricane Database), JMA, and JTWC (Joint Typhoon Warning Center) forecast/best track data sets for all named storms and is available for all landfalling TCs since 2004, which would reduce challenges that arise when applying an index on a global or a regional scale. Furthermore, the relationship between *R*_*50*_ and storm surge forecasting has been discussed in many prior studies^21,29,40,41^.

*V*_*ref*_, *R*_*ref*_, and *S*_*ref*_ are reference constants and are defined as 50-kt, 95 nm (historical mean *R*_*50*_ in Japan^42^), and 35 km/h (historical mean translation speed of TCs impacting in Japan^42^), respectively. L* is chosen to be 40 km to make SSHPI roughly equal in magnitude to the peak storm surge height. These reference values are used for normalization that prevents the index values from being biased toward extreme values. The structure of Eq. (4) does not represent the orderly contribution of each SSHPI component in generating surge hazards.

It is noted that a stationary or very slow-moving TC (i.e., *S* = 5 km/h) would result in very low SSHPI numbers (using Eq. (4)) on the open coast and extremely high numbers in semi-enclosed bays (vice-versa). TC with a very large size (i.e., *R*_*50*_ = 170 nm) would also result in very high SSHPI numbers (vice-versa). Although such TCs are infrequent in Japan but can sometimes occur elsewhere. The form of Eq. 4 without upper and lower bound of TC size and forward speed would probably not be a good representative for the surge hazard poses by such unusual TCs. Therefore, we limit 0.5 ≤ $(\frac{{R}_{50}}{{R}_{ref}})$  ≤ 1.5 and 0.5 ≤ ${(\frac{S}{{S}_{ref}})}^{a}$  ≤ 1.5 in Eq. (4) when *S* and *R*_*50*_ are exceptionally large or small. This upper and lower bound of TC size and forward speed will prevent discrete jumps in SSHPI numbers.

The linear dependence of SSHPI on TC forward speed is twofold. First, in semi-enclosed bays, the effective cross-shore shallow area over which TC winds act is larger; the contribution of wind stress tends to be more pronounced in the bay than the open coastlines due to a shallower depth^43^. Also, the time scale for mass redistribution (to generate a sea surface slope) within the shallow and geometrically complex estuaries is on the order of hours and longer than along the open coasts^19^. Thereby, with cross-shore wind stress components, a slower TC has more time to interact with the seawater and pushes more into shallow areas of a bay. Consequently, surge height begins to fully develop (or mature), causing a large sea-level gradient between the upper and lower bay. Thus, we set *a* = − 1 for semi-enclosed bay and by construction, large (i.e., *R*_*50*_ > 95 nm), but slow-moving (i.e., *S* < 35 km/h) and intense (i.e., *V*_*max*_ > 50-kt) TCs will generate greater storm surge potential.

On the contrary, in the open coastlines, it is plausible that a fast-moving TC would energize a shelf wave and cause an increased storm surge because the TC translation speed tends to coincide with the long-wave propagation speed^44^. This mechanism could be partially explained by Proudman’s linear theory^45^, which showed that storm surges could be amplified when the TC translation speed was similar to the propagation speed of the long wave ($\surd gh$). With *a* set to 1, fast-moving (i.e., *S* > 35 km/h) storms will generate larger SSHPI. It should be noted that we did not directly consider inverse barometer effect (IBE) and the influence of TC approach angle, waves, and astronomical tide to keep SSHPI simple. Thus, SSHPI will tend to underestimate/overestimate total surge height somewhat for some TC events. The limitation is particularly relevant in open coasts, where wave set-up and IBE are often the dominant drivers behind storm surge and coastal flooding^46–48^. Furthermore, a very steep coast (i.e., *L*_*30m*_ = 0.5 km) would result in a low SSHPI number (using Eq. 4), a strong TC could, however, still cause significant storm surge. Lastly, the SSHPI computes peak surge hazard potential at points in the coasts, but the prediction of seawater inundation in coastal land areas remains beyond its scope.

## Data and method

We use JMA best track data archives from 1978 to 2019. The best track data acquired during the pre-satellite era (i.e., before 1978) contain inhomogeneities and large uncertainties in the data quality^49,50^ and therefore were ignored. JMA best-track data contain 6-hourly TC central position, intensity, size, and forward speed information. TCs were selected based on the following criteria: (a) TCs that made landfall in Japan; (b) TCs that incurred a minimum of 40 cm of storm surge, and (c) TCs that had intensity, size (*R*_*50*_), and forward speed information (during landfall time frame) were available. The choice of 40 cm is to evaluate storm surge index accuracy for more severe storm surge events.

**Figure 1:**
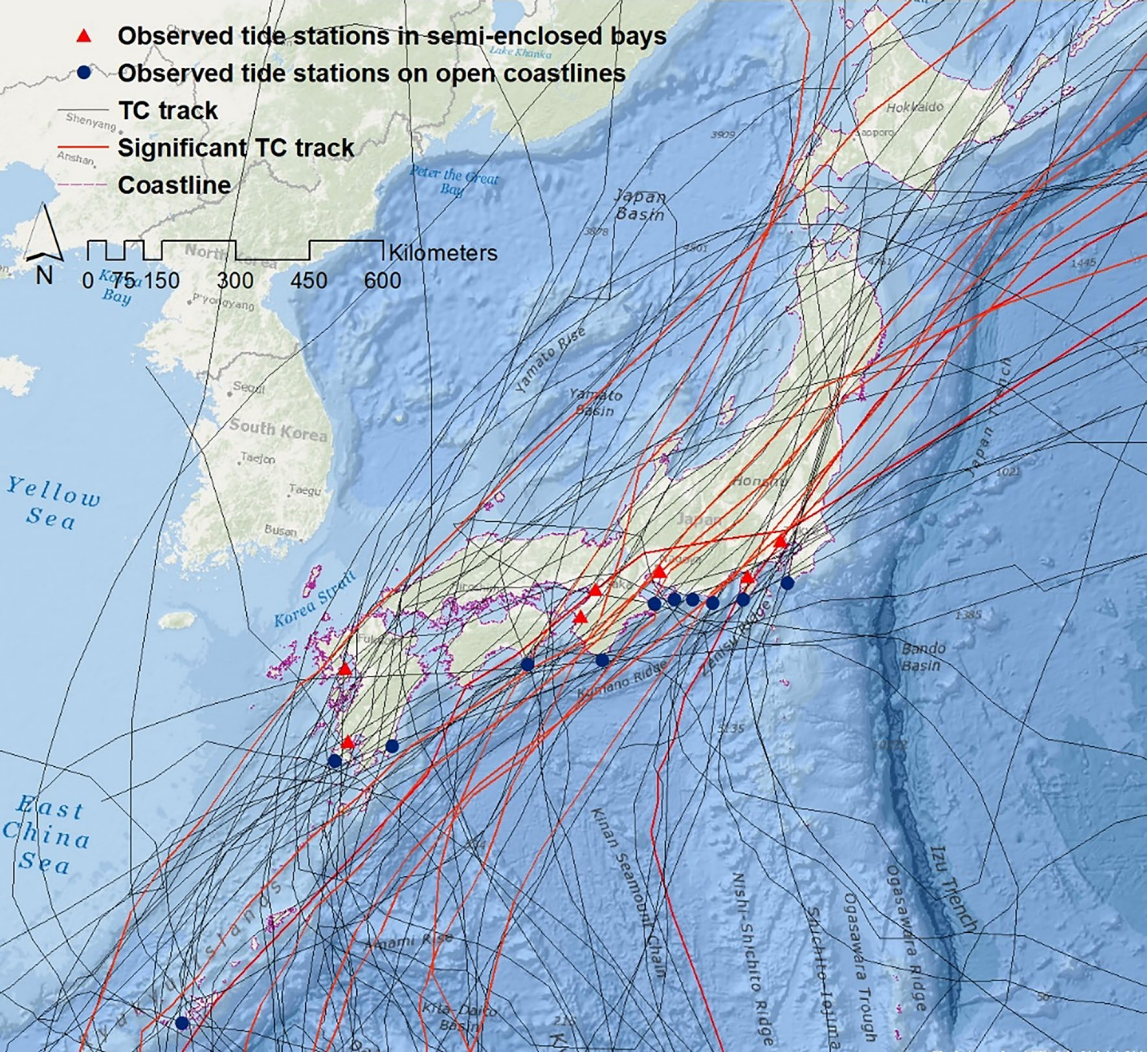
Best track for historical TCs^42^ making landfall (based on the criteria used in this study) during 1978–2019 over the four major Japanese islands (Honshu, Shikoku, Kyushu, and Okinawa). Two different symbols indicate seven stations in semi-enclosed bays and 11 stations in open coasts. Map is created using ArcMap (v. 10.2).

Based on the above three criteria, 51 TCs were selected for analysis (Fig. 1). Among them, 15 made landfall directly over open coastlines (directly facing the Pacific Ocean), while seven directly hit bay areas (regions surrounded by two land areas that form a concave-shaped coastline). The remaining 29 TCs made landfall between open coastlines and bay areas and impacted both regions. Of the 51 TCs, 19 (10) TCs impacted more than one tide station located on the open coastlines (bay areas). As a result, there were 70 and 47 available storm surge cases for open coasts and bay areas, respectively and thus, 117 cases total. Since the sample size (n) is small and excludes several significant surge events that occurred before 1978, such as TC Vera (1959) and Nancy (1961), these may influence the overall statistics (i.e., overestimate/underestimate significant surge events) shown in the present study. Nonetheless, the period from 1978 to 2019 is the longest period covered by the JMA best track with uniform data quality.

**Figure 2:**
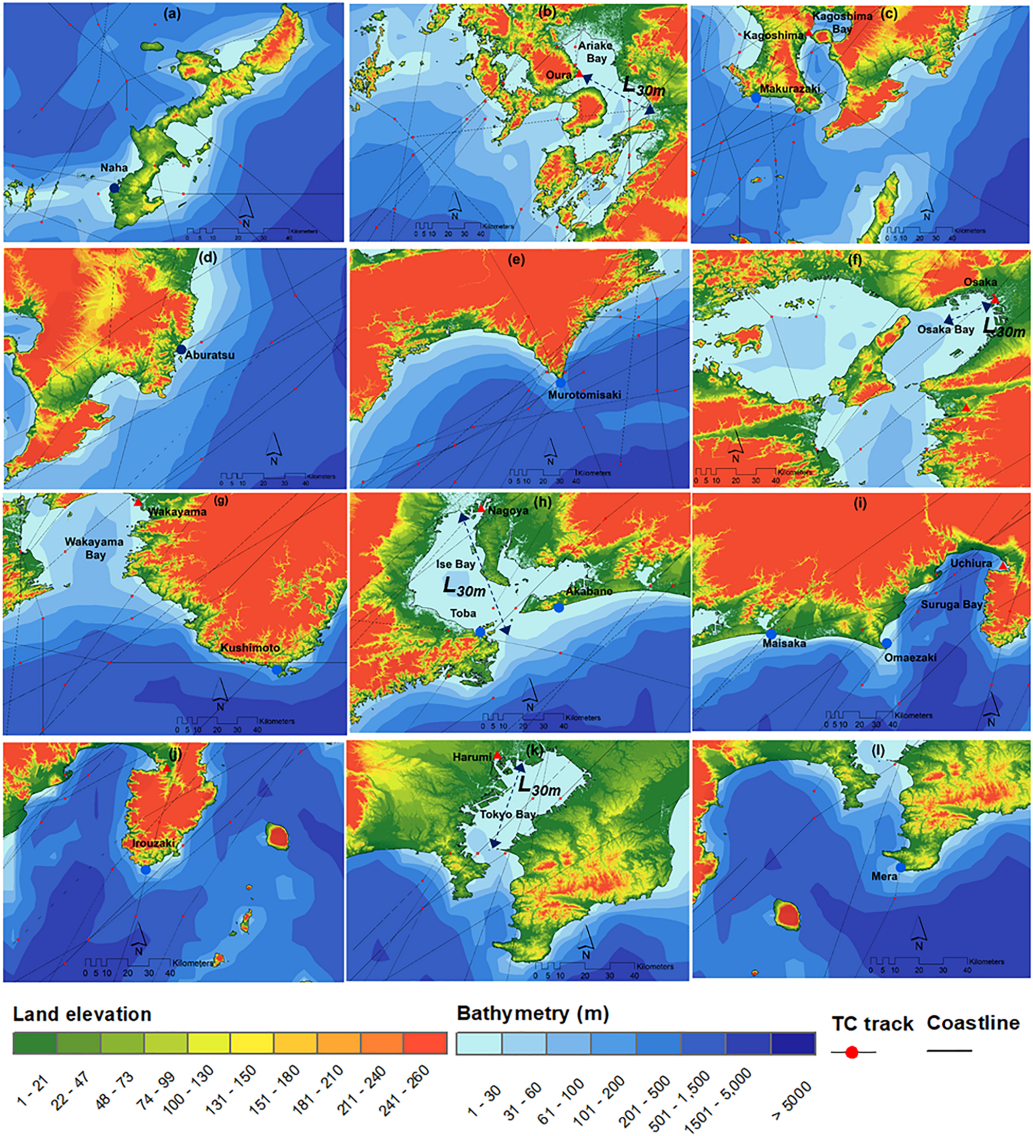
Location map of observed tide stations **(a)** Naha, Okinawa island; **(b)** Oura, Kyushu island; **(c)** Makurazaki and Kagoshima, Kyushu island; **(d)** Aburatsu, Kyushu island; **(e)** Murotomisaki, Shikoku island; **(f)** Osaka, Honshu island; **(g)** Wakayama and Kushimoto, Honshu island; **(h)** Toba, Nagoya, and Akabane, Honshu island; **(i)** Maisaka, Omaezaki, and Uchiura, Honshu island; **(j)** Irouzaki, Honshu island; **(k)** Harumi, Honshu island; **(l)** Mera, Honshu island. All maps are created using ArcMap (v. 10.2).

Table 2 shows the predictors used in the SSHPI. For sensitivity tests, we also use the radius of tropical storm wind speed (30 kt, *R*_*30*_) instead of *R*_*50*_. Note that JMA provides two types of wind radius information: the longest and shortest radius^42^. The translation speed at time *T* is calculated with the TC central positions at *T* and *T* – 6 h. For cases in which translation speed, intensity, and *R*_*50*_ data was unavailable immediately before TC landfall time, those data were obtained via linear interpolation of the available data at two neighboring positions (nearest before and after landfall). The bathymetry data over the target area was obtained from the Japan Coast Guard^51^. GIS environment was used to measure the closest horizontal distance between each selected tide station and 30 m depth contour (*L*_*30m*_) (i.e., Fig. 2b,f,h,k).

**Table 2:** TC databases and their scope and limitations based on coverage, resolution, and availability.

Database	Type	Resolution		Unit	Data range
		Temporal	Spatial		
TC 10 min—sustained wind speed^42^	Best track	6 hourly	‒	kt	1978–2019
TC central sea-level pressure^42^	Best track	6 hourly	‒	hPa	1978–2019
TC Size (radius of 50-kt wind, radius of 30-kt wind)^42^	Best track	6 hourly	‒	nm	1978–2019
TC Forward speed^42^	Best track	6 hourly	‒	Km/h	1978–2019
Coastal bathymetry^51^	Gridded bathymetry data	‒	500 m	m	24°N—46°N 122°E—148°E
Observed storm ride^52^	‒	1 hourly	‒	cm	1978–2019
Predicted astronomic tide^53^	‒	1 hourly	‒	cm	1978–2019
Observed storm surge^52^	‒	1 hourly	‒	cm	1978–2019

The storm surge heights recorded at 18 JMA-operated tidal stations^52^ were used to estimate the peak storm surge for each TC. These stations satisfied the following criteria: (a) located on open coastline or in a bay; (b) JMA predicted astronomical tide data^53^ were available; (c) elevation of the observation reference plane and the astronomical tide table reference plane were available; (d) fell right side of a selected TC track and located within the range of *R*_*50*_ (during TC landfall time frame); and (e) no data were missing when a TC traversed the station. Eleven stations (Naha, Makurazaki, Aburatsu, Murotomisaki, Kushimoto, Toba, Akabane, Maisaka, Omaezaki, Irouzaki, and Mera) were selected as representative observatories for storm surges on open coastlines and seven (Oura, Kagoshima, Osaka, Wakayama, Nagoya, Uchiura, and Harumi) were selected as representative observatories for storm surges in semi-enclosed bays. Figure 2 provides details of the selected tide stations. Sea surface anomalies were assumed to be the storm surge magnitude, and they were estimated by deducting the predicted astronomical tide from the observed storm tide.

## Analysis of the new index

### The performance of SSHPI

**Figure 3:**
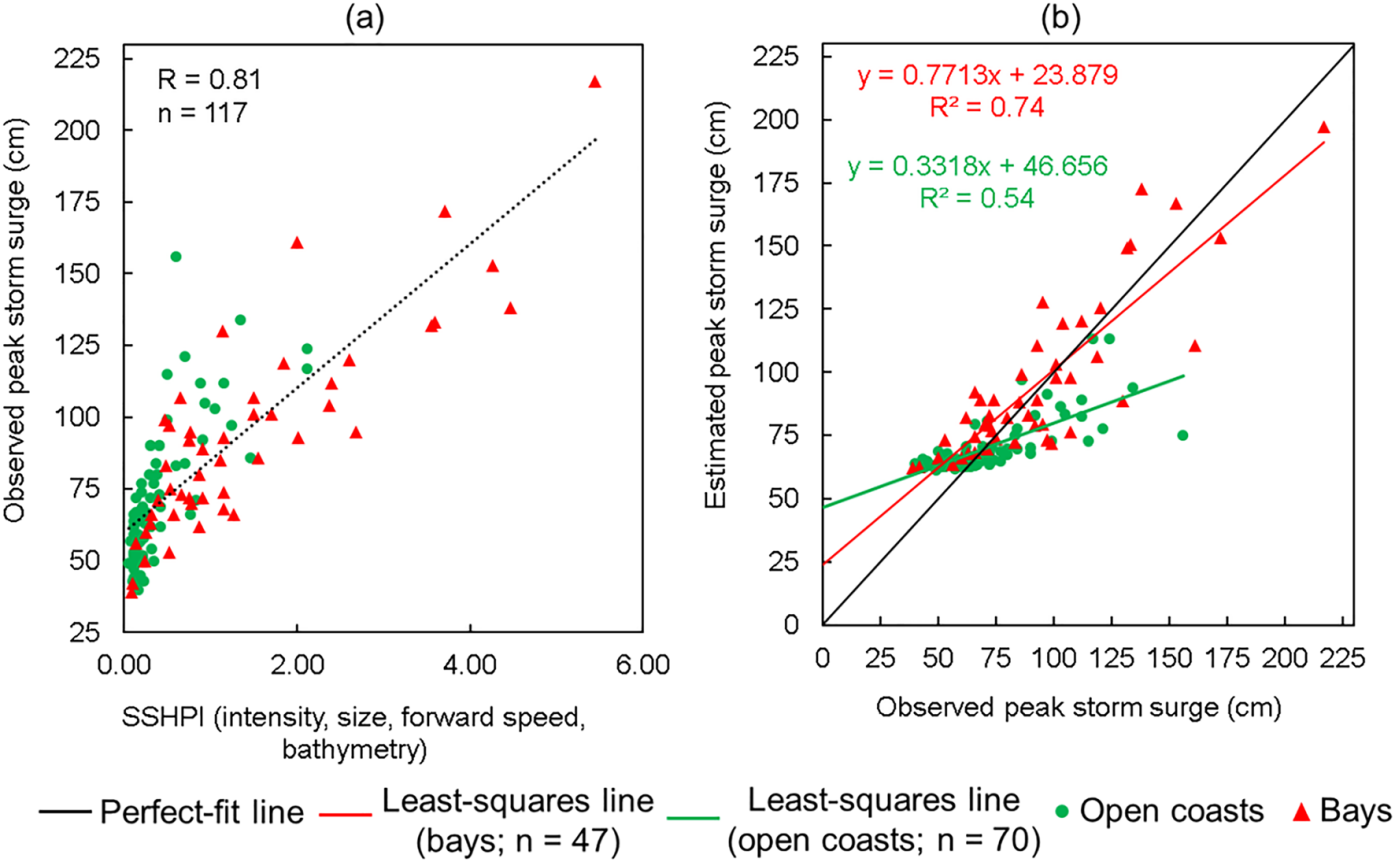
**(a)** Observed TC peak surges (ξ) versus SSHPI (Eq. 4) as a function of intensity, size, forward speed, and bathymetry. Dashed line shows the correlation gradient of the respective surge (ξ) and SSHPI; **(b)** Scatter plot of estimated (Eq. 5) and observed peak storm surge heights at selected stations.

The SSHPI is positive proportional to the peak surge height. Fig. 3a shows that the Pearson correlation statistic (R) between SSHPI and observed surge height is 0.81 (p < .01 at 95% confidence level) and explains ~66% of the observed variance. These statistics are comparable to storm surge estimation obtained from full physical numerical surge models^54,55^ which comprehensively account for coastal surge dynamics.

In the sensitivity test where we replaced *V*_*max*_ with *∆p* as an intensity metric in Eq. (4). The resulting R statistic between SSHPI and observed surge height remains almost unchanged (not shown); this may be because that the *V*_*max*_ and MSLP are strongly associate with each other (R = 0.83, p < 0.01; not shown) as discussed in previous studies^56–58^. Correlation corresponding to the *R*_*30*_, was also calculated to explore whether any other definition of size metric would provide a better correlation than *R*_*50*_. The correlation statistic did not vary markedly (R = 0.79, p < 0.01) from the statistic presented in Fig. 3a. The performance of SSHPI is insensitive to *V*_*max*_ vs. MSLP and *R*_*50*_ vs. *R*_*30*_ and thus, the alternative parameters (MSLP and *R*_*30*_) could potentially be used at a basin for a period where *V*_*max*_ and *R*_*50*_ are unavailable.

Next, in an attempt to make sure that the significant storm surge events did not skew the Pearson correlation analysis, we performed additional correlation analysis for SSHPI but excluding the three highest storm surge events caused by Typhoon Mireille (1991), Typhoon Flo (1990), and Typhoon Jebi (2018). While the correlation coefficient decreases slightly, from 0.81 to 0.75 (p < 0.01), it remains significant and strong.

A least squares-fit between surge height and SSHPI (Fig. 3a) gives an empirical relationship between the two:

5 $$ \xi_{{{\text{est}}}} = { 25}.{14 } \times {\text{ SSHPI }} + { 6}0.0{5} $$

We emphasize that we favor predicting SSHPI—an index-based parameter, instead of a direct surge measure to mimic SSHWS for easy public communication. Still, Eq. (5) provides a path to convert SSHPI to peak surge height (*ξ*_*est*_). The scatter plot (Fig. 3b) illustrates that the *ξ*_*est*_ also correlates well with the observations, similar R statistic as for SSHPI. This is expected since they are linearly related to each other (not shown). Fig. 3b exhibits majority of the *ξ*_*est*_ are close to the observations. Root-mean square error (RMSE) in the estimated surge is ±18.09 cm, smaller than JMA’s numerical storm surge prediction model errors^47^ (±50 cm). It needs to be noted that JMA applies TC wind and pressure field to the numerical storm surge model as external forcing. Their TC model diagnoses wind and pressure fields using the necessary input of forecast values, including the TC center location, minimum pressure at the center, *V*_*max*_, *R*_*50*_ (if present), and the radius of 1000 hPa^46^. The difference in RMSE between SSHPI and JMA numerical model predicted surges is primarily because meteorological inputs for current SSHPI analysis are from best track data (post-processed), while JMA evaluated their surge model applying forecasted products during the period of 2015-2017^47^. Nonetheless, operational forecasts of TC have been improving gradually, the performance of SSHPI showed in this study may not be affected significantly in forecast settings (considering SSHPI inputs are from TC forecasted advisories). The performance of the Eq. (5) for estimating storm surge height decreases (the least-squares line diverges from the best-fit line) for more significant surge cases. The underestimation of significant surge levels may be due to the simplified physics considered in SSHPI and/or the observational errors.

Given the limitation that *R*_*33*_ used in other indices is not included in the JMA best track data set, therefore, it is not possible to compare the performance of SSHPI with other surge indices (i.e., HSI^32,33^, SS^34^) for TCs in Japan. However, we attempted to apply SSHPI for the US significant surge cases (see Supplementary Information for data details and Supplementary Fig. S1) and compared the results with HSI (Eq. 1) and SS (Eq. 3). The analysis (see Supplementary Fig. S2) shows that SSHPI appropriately reflects the relative magnitude of expected surge (R = 0.85; p < 0.01).

Next, we use the probability of detection (POD)^59^, the false alarm ratio (FAR)^59^, and the bias score (BS)^59^ to assess the accuracy of the SSHPI quantitatively. Their mathematical forms are:

6 $$\mathrm{POD}=\frac{(hits)}{\left(hits\right)+(misses) };0 \le \mathrm{ POD }\le 1;\mathrm{ the\,\, perfect \,\,score\,\, is\,\, }1$$

7 $$\mathrm{FAR }= \frac{(false\, alarms)}{\left(hits\right)+(false\, alarms) }; 0 \le \mathrm{ FAR }\le 1;\mathrm{ the\,\, perfect\,\, score\,\, is\,\, }0$$

8 $$\mathrm{BS }=\frac{\left(hits\right)+(false alarms)}{\left(hits\right)+(misses) }; 0 \le \mathrm{ BS};\mathrm{ the\,\, perfect\,\, score\,\, is\,\, }1$$

**Figure 4:**
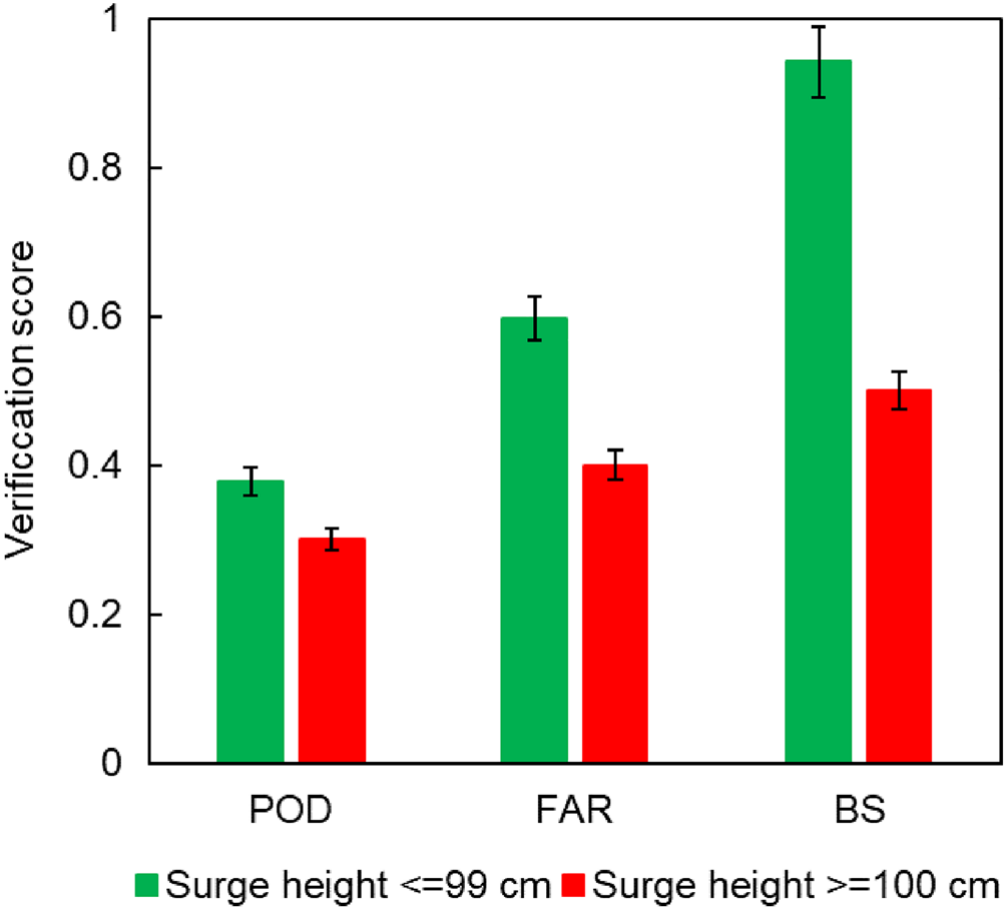
Verification scores for SSHPI estimated storm surges. Error bars show two-sided 95% confidence intervals.

All three scores (Fig. 4) suggests that the SSHPI performs better for events with smaller surge heights (i.e., ≤ 99 cm) than for those with larger storm surges (i.e., ≥ 100 cm). For example, the BS score for minor surge events is close to 1, while it is 0.5 for significant surge events. The BS and FAR scores indicate that SSHPI is unlikely to overestimate the peak surge height.

### SSHPI dependency on the predictors

**Figure 5:**
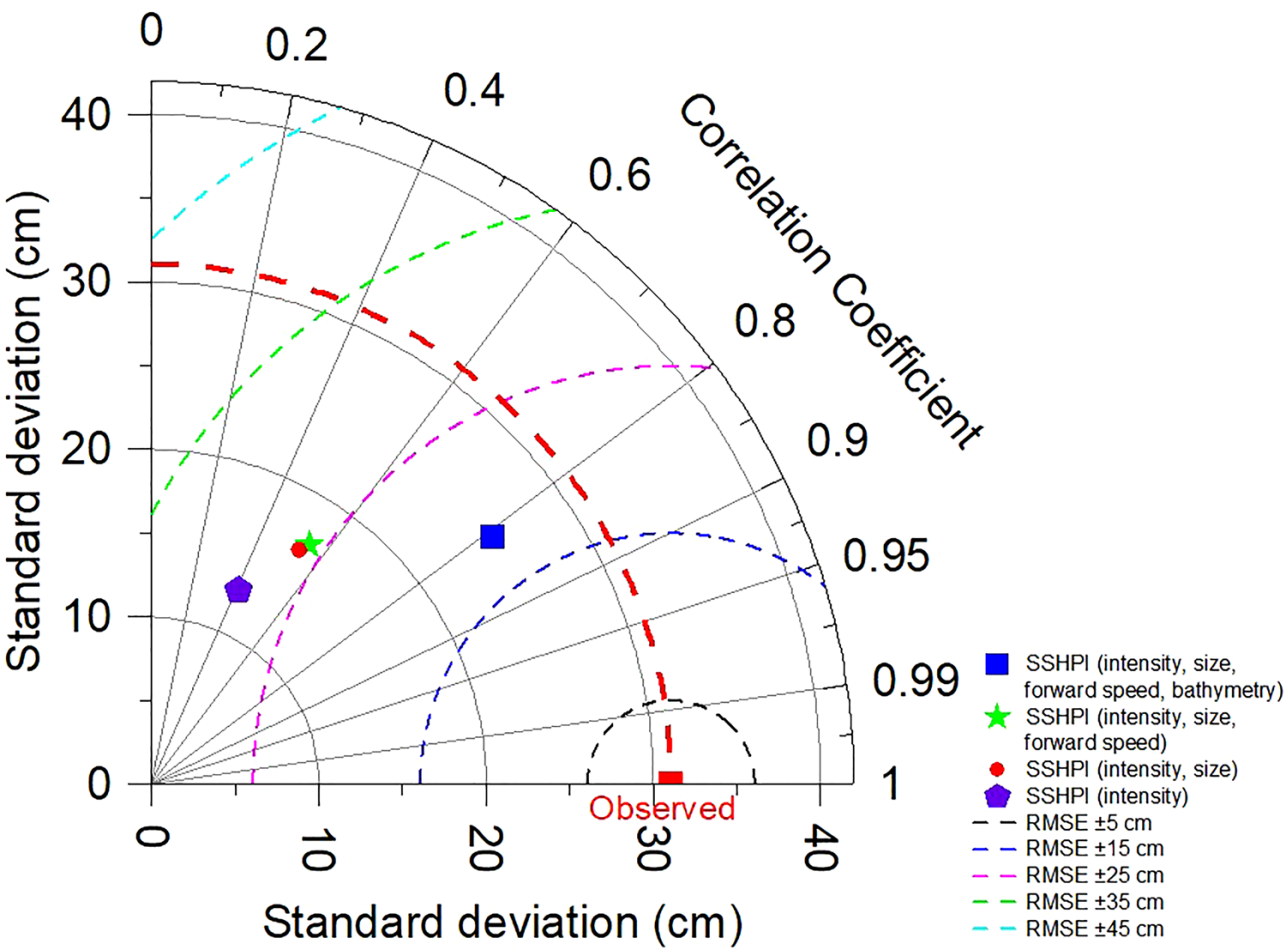
Taylor diagram describing the performance of SSHPI by comparing with the reduced versions of SSHPI. The azimuthal angle represents correlation, the radial distance the standard deviation, and the semicircles centered at the “Observed” marker the root mean square error. The red dashed line constitutes the standard deviation of observed peak storm surge heights (n = 117).

To better understand the dependency of the SSHPI on its predictors, statistical measures (standard deviation (*σ)*, RMSE) and correlation analyses were conducted using the versions of SSHPI that do not include all of the predictors, i.e., reduced versions of SSHPI (SSHPI (intensity); SSHPI (intensity, size); SSHPI (intensity, size, forward speed)). Figure 5 shows that a TC intensity-based scale, which is similar to the traditional SSHWS, is only weakly correlated with observed surge (R = 0.41; (p < .01)), largest RMSE (±28.22 cm) and different variability (*σ* = 12.7 cm) than the observations (*σ* = 31.06 cm). Gradual improvements are apparent as the TC parameters (size and forward speed information) are added to the surge indices and they have R = 0.50 (p < .01) and R = 0.55 (p < .01), respectively. It is worth noting that the Pearson correlation statistic for SSHPI (R = 0.81, p < .01) shows significant improvement over all three of the surge indices and the differences are significant at the 5% level. Comparing to other indices, SSHPI has similar variability (*σ* = 25.19 cm) as the observations has, the highest correlation, and the least RMSE (±18.09 cm). A similar result can also be confirmed utilizing principal component analysis.

**Figure 6:**
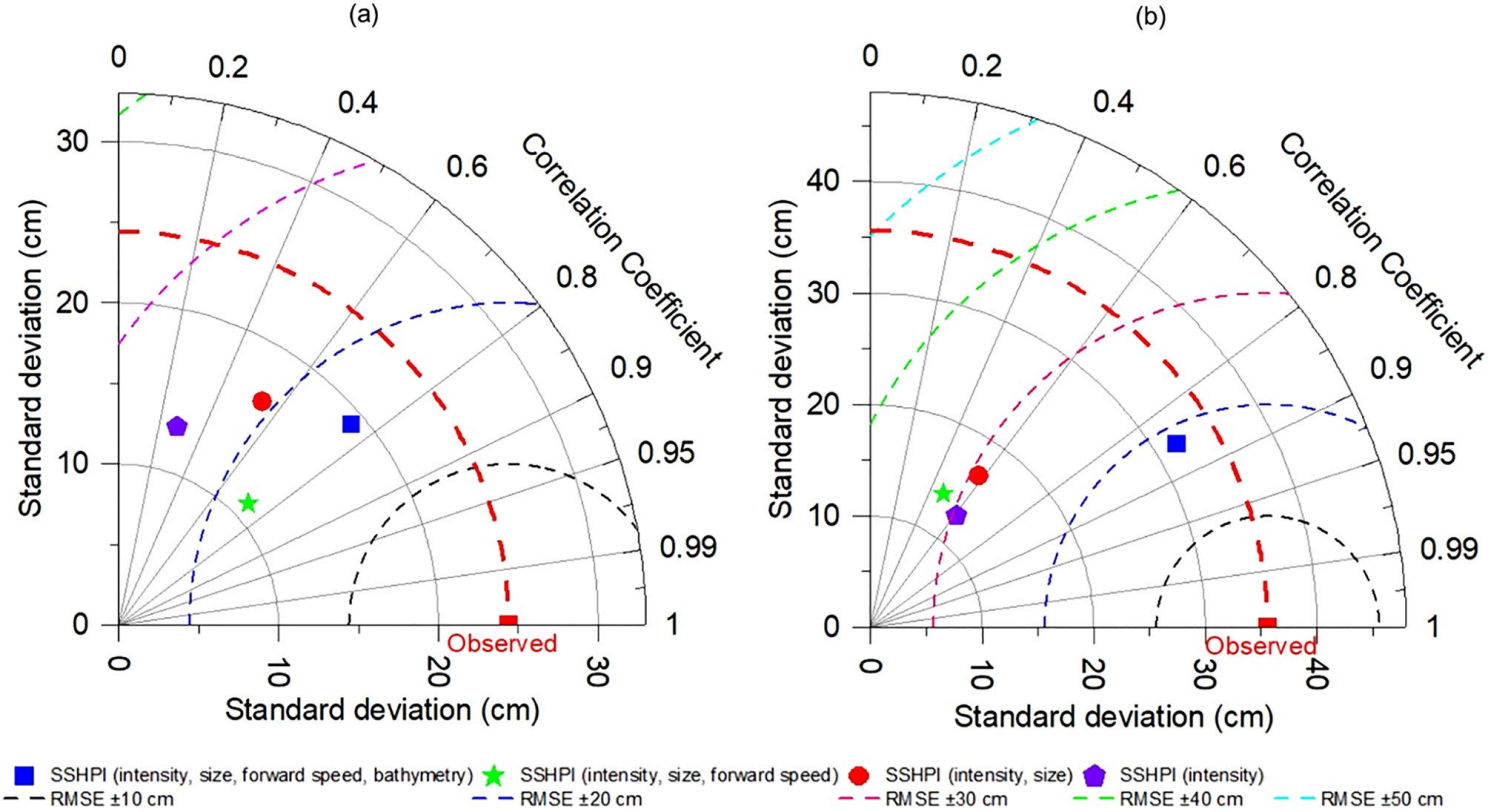
Taylor diagram describing the performance of SSHPI with varying coastal geometry **(a)** open coast (n = 70); **(b)** semi-enclosed bays (n = 47). The azimuthal angle represents correlation, the radial distance the standard deviation, and the semicircles centered at the “Observed” marker the root mean square error. The red dashed line constitutes the standard deviation of observed peak storm surge heights.

Batstone et al.^60^, Maskel et al.^61^, and Lyddon et al.^62^ have noted that surge predictions are complicated in estuaries or bays due to its topographic features, shape, and tide-river flow interactions. In other words, one can expect that the dependence of SSHPI and the predictors may vary with the coastal geometry. Thus, here we repeat analyses for Fig. 5 but using data for open coasts (n = 70; Fig. 6a) and semi-enclosed bays (n = 47; 6b), respectively. Correlation between storm surge height and SSHPI in both cases are still much higher than those between *V*_*max*_ and storm surge height and the RMSE is much lower. In particular, *V*_*max*_ substantially underperforms in predicting surge potential along the open coastlines (Fig. 6a). It is reasonable as the wind set-up is generally quite limited in open coasts (due to steep coastline) and the IBE, wave set-up, and ocean currents dominate surge hazard^46–48^. Nonetheless, adding TC size and forward speed largely increases the correlation and reduces the RMSE and helps to explain ~ 50% more of the observed surge variance (Fig. 6a). In the semi-enclosed bay areas, adding bathymetry information improves the surge variance by ~ 37% (Fig. 6b). The bay areas are typically characterized by shallow water (i.e., less than 30 m) which largely modulates local storm surge profiles^63^. It needs to be noted that wind set-up and bathymetry together can explain ~ 59% surge variance in semi-enclosed bays, the contribution of TC size and forward speed, however, is not negligible (improves by ~ 15%; see Supplementary Fig. S3). Overall, these results support the previous studies^14,21,28,32,34^ that storm surge potential is more than a function of the intensity of the TC, while surge is potentially driven by the size, forward speed, and amplified by TCs making landfall in shallow coastal areas such as bays.

### Hindcasting major storm surge events

**Figure 7:**
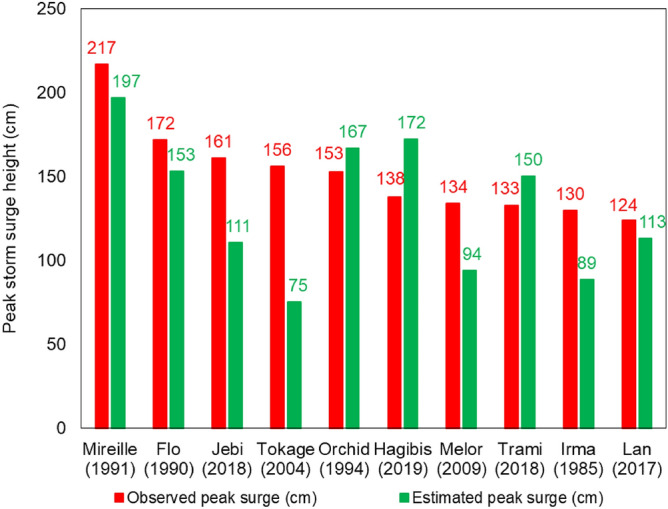
Top ten storm surge events in Japan since 1978 and the estimated peak surge using SSHPI (Eqs. 4 and 5).

We next examine the accuracy of SSHPI in predicting major storm surge events using two criteria: (a) estimating the largest ten storm surge events (using Eqs. 4 and 5) impacting the Japanese coastlines over the last 42 years (tracks are shown in Fig. 1) and (b) storm surges (n = 37) caused by TCs that had wind speeds ≥ 80-kt during the landfall time frame. Figure 7 shows that for the two largest surge events caused by TC Mireille (1991); TC Flo (1990), the SSHPI is larger than 3.7 (Fig. 3a), and the estimated surge heights are 197 cm and 153 cm (Eq. 5), both are close to the observed value, 217 cm and 172 cm, respectively. For TC Jebi, the SSHPI value based on meteorological observations is 2.01, which suggests a surge level of 111 cm. The observed peak surge for Jebi is 161 cm (at Osaka). In the case of Jebi, surge was largely contributed to by high waves^64–66^, which is not considered in the SSHPI. The SSHPI also underestimates the observed value of Tokage in 2004. This may be because the observation site (Murotomisaki) is located on a steep-slope coast facing the open sea, where the combined influence of wave-set up and IBE is large. Overall, the estimated major storm surge events agree well with the recorded surge data, although RMSE increases to ±38.7 cm for this set of surge events than the RMSE (±18.09 cm) for all storm surge cases.

**Figure 8:**
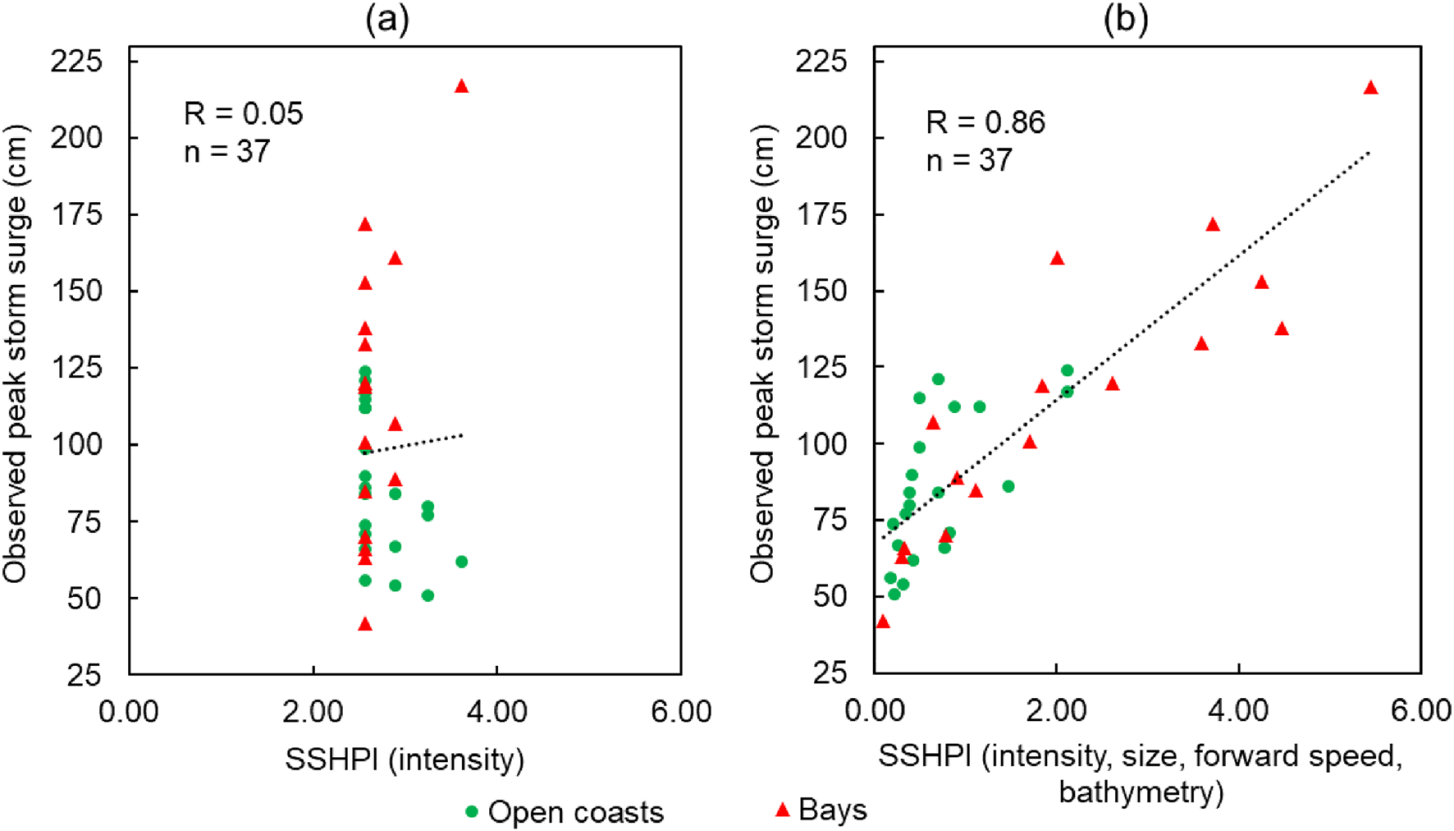
TC intensity-based (*V*_*max*_ ≥ 80-kt, during landfall time frame) definition of major storm surges versus **(a)** SSHPI as a function of intensity; **(b)** SSHPI as a function of intensity, size, forward speed, and bathymetry; Dashed line shows the correlation gradient of the respective surge and SSHPI.

If we use TC intensity-based (*V*_*max*_) definition of major storm surge events, Fig. 8 shows SSHPI can predict storm surge hazard potential with much greater certainty (R = 0.86, p < 0.01; Fig. 8b) than the intensity-based scale (R = 0.05, p > 0.05; Fig. 8a). SSHPI appropriately assigns relatively higher values for the major surge events. For the storm surge records more than 115 cm, the SSHPI is roughly 2.4 or larger. It is worth noting in Fig. 8a that storm surge magnitudes significantly differ under the similar intensity category of historical TCs and surge has been overstated in most of these cases. In the present storm surge dataset since 1978, there have been a total of 37 events caused by TCs that had the wind speed ≥ 80-kt during the landfall time frame (Table S1). A total of 30 storm surge events including four major storm surge events (listed in Fig. 7) including Flo (1990), Mireille (1991), Orchid (1994), and Lan (2017) can be better predicted with the SSHPI based scale than with the TC intensity-based scale (Table S1). It needs to be noted that except TC Mireille (category 2, *V*_*max*_ = 95-kt), the rest were simply categorized as category 1 hurricanes (64-kt ≤ *V*_*max*_ ≤ 82-kt) during the landfall time frame in SSHWS scale.

While the SSHPI appropriately reflects the relative magnitude of historical surge events using JMA best track data, questions regarding the applicability of SSHPI in operational forecast settings may arise. As forecasted advisories for historical TCs in Japan are publicly unavailable, further study is required. Moreover, transdisciplinary efforts and collaboration between operational agencies and academics are required before SSHPI could be suitable for an operational forecast communication product. Nonetheless, we attempted to utilize SSHPI in the NHC forecast settings to quantify surge hazard for Hurricane Katrina around the Mississippi coasts, US (see Supplementary Fig. S4). The results show that SSHPI accurately characterizes the surge hazard caused by Katrina a day before the impacts were realized. It is also noticeable that SSHPI can describe the distribution of peak surge hazards around the landfall point at a large spatial scale.

## Summary and conclusion

Here we demonstrated the development of a new storm surge hazard potential index (SSHPI) for quantifying/categorizing TC induced surge events in Japan. When applied retrospectively, it explains ~66% of the observed variance. A fundamental difference between the SSHPI and existing indices is that it considers coastal geometry and storm forward motion speed in surge estimates. Using reduced-versions of SSHPI, we found that while surge estimation derived from using only intensity and storm size information provides less information on the overall surge hazard than does full form of SSHPI.

SSHPI utilizes the most common and readily available TC meteorological parameters, coastal geometry, and bathymetry information and thus can, hopefully, be applied to surge extremes produced by TCs in other basins (i.e., North Atlantic, North Indian Ocean). However, in that case, the index values and its associated hazard potential showed in this study likely have to be revised, as the surge hazard potential may be different in other basins. For instance, SSHPI values larger than 4.0 are representative of extremely dangerous for Japan, however, it may not necessarily constitute similar surge hazard potential in areas characterized by a large continental shelf or shallow ocean waters.

SSHPI can predict hazardous surge events for Japan well and perform better for surge events caused by TCs with landfall intensity larger than 79-kt. It also has the advantage of providing an instantaneous measure of a TC’s surge potential without heavy computational effort. Thus, it could be of utility to the general public for pre-TC measures and post-TC analysis. SSHPI values could be utilized in countries at risk of storm surge but have no access to advanced surge models. Furthermore, it can explain temporal variations in surge events on global, regional, and local scales as it considers climatological variables of a TC.

It should be noted that SSHPI proposed in this study is largely dependent on the quality of the forecasted track information. The correlation statistics for SSHPI shown in this study will improve as the uncertainties^67^ associated with TC forecasted track information get smaller. The empirical equation (Eq. 5) used in this study may not always provide an accurate prediction of peak storm surge as it does not reflect the complete surge characteristic of a specific site. Therefore, it is advisable to develop tidal observatory/site specific prediction equation using SSHPI data for the historical period. Furthermore, it would be interesting to determine whether additional variables (i.e., IBE, TC approach angle, landfall location, continental slope, gust wind, wave set-up) could or should be included in the SSHPI. Risk communication efforts in various disasters have shown that a categorization-based severity index (i.e., potential to damage), similar in philosophy to SSHWS could provide valuable input on public risk perception^17^. Ultimately, we would like to use SSHPI as SSHWS which provides public an intuitive understanding of the surge damage. Thus, the next step of this work includes connecting SSHPI to the losses, i.e., the actual risk, exploring effective communication methods through categorization, graphical and verbal techniques, which requires interdisciplinary efforts and collaboration between operational agencies and academics. Lastly, a global database of SSHPI and surge records would largely increase the usage of the SSHPI.

## Supplementary Information

Supplementary Information.
