# Supplementary material for: A new tropical cyclone surge index incorporating the effects of coastal geometry, bathymetry and storm information
Source: Sci Rep. 2021 Aug 18;11:16747. doi: 10.1038/s41598-021-95825-7 (PMC8373937; doi:10.1038/s41598-021-95825-7)
Supplement: Supplementary file 1 — Supplementary Information. [file 41598_2021_95825_MOESM1_ESM.docx]

# A new tropical cyclone surge index incorporating the effects of coastal geometry, bathymetry and storm information

# Md. Rezuanul Islam^1,*^, Chia-Ying Lee^2^, Kyle T. Mandli^3^, and Hiroshi Takagi^1^

^1^ Department of Transdisciplinary Science and Engineering, School of Environment and Society, Tokyo Institute of Technology, Tokyo 152-8550, Japan

^2^ Lamont-Doherty Earth Observatory, Columbia University, Palisades, New York 10964, United States

^3^ Department of Applied Physics and Applied Mathematics, Columbia University, New York 10964, United States

^*^islam.m.ac@m.titech.ac.jp

**Supplementary information**

**SSHPI performance analysis for the US storm surge cases**

Although this study focuses on TC-induced storm surge cases in Japan, we also analyzed SSHPI performance for the US storm surge events. Similar TC selection criteria discussed in the “data and method” section and adding one more condition: TCs that have *R_33_* during landfall time frame were used to select TCs in the US. In this manner, a total of 25 TCs were selected for storm surge analysis (Fig. S1). We use NHC best track data (Atlantic HURDAT2) archives^68^ from 2004 to 2019. The best track data before 2004 does not contain *R_50_* data and therefore were ignored. A total of 30 storm surge cases were analyzed, with 19 and 11 available storm surges on open coasts and bay areas, respectively. Figure S1 shows 25 tidal stations^69,70^ that were used to estimate the peak storm surge for each TC. Among them, 16 stations were selected from open coastlines (Bob Hall Pier: Corpus Christi, Cameron, Destin: FL, Duke marine lab, Everglades City, Extreme SW FL, Key West, Pass Christian, Pilots Station East: S.W Pass LA, S Padre Is Port, S Padre Island, Sandy Hook, Sanibel and Estero, Springmaid Pier: SC, Trident Pier, and USGS Station Hatteras) and the other nine stations were located in bays (Apalachicola: FL, Bay Gardene, Chambers Country, Coast Guard Sector Mobile, Mckay bay Entrance: FL, SE-LA-MS Lakes, Shell Beach: LA, Ship John Shoal: NJ, and The Battery: NY).

Figure S2 shows that SSHPI can explain ~72% (R = 0.85, p < .01) of observed surge variance for the given set of data in the US. The efficiency of SSHPI described for Japan surge cases remains identical for US surge events as it has reasonably similar variability (σ = 1.41 m) compared with the observations, the highest correlation, and the least RMSE (±0.87 m).


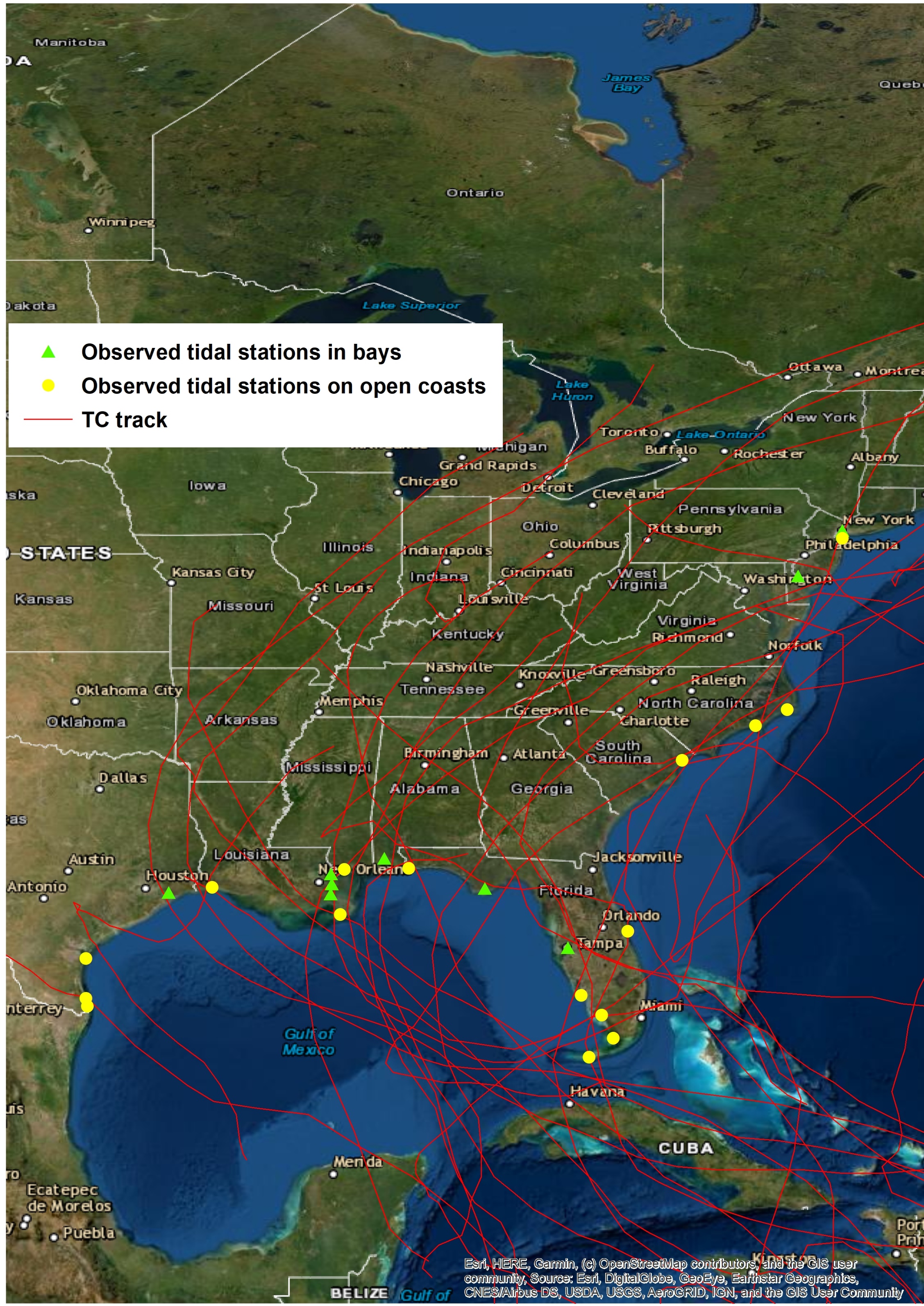


**Figure S1.** Best track for historical tropical cyclones^68^ making landfall (based on the criteria used in this study) during 2004–2019 over the US based on data from the NHC. Two different symbols indicate nine stations in bays and 16 stations on open coasts. Map is created using ArcMap (v. 10.2).


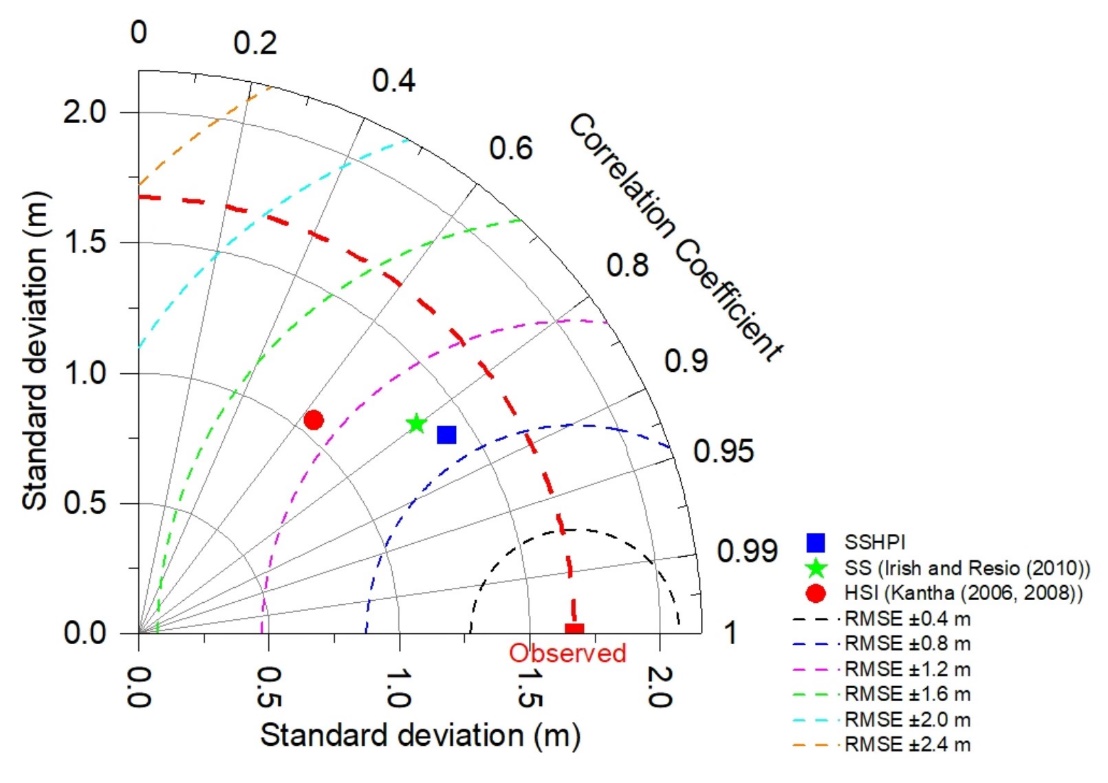


**Figure S2.** Taylor diagram describing the performance of SSHPI by comparing with the SS^34^ and HSI^32,33^. The azimuthal angle represents correlation, the radial distance the standard deviation (σ), and the semicircles centered at the “Observed” marker the root mean square error. The red dashed line constitutes the standard deviation of observed peak storm surge heights.

**SSHPI dependency on the predictors**


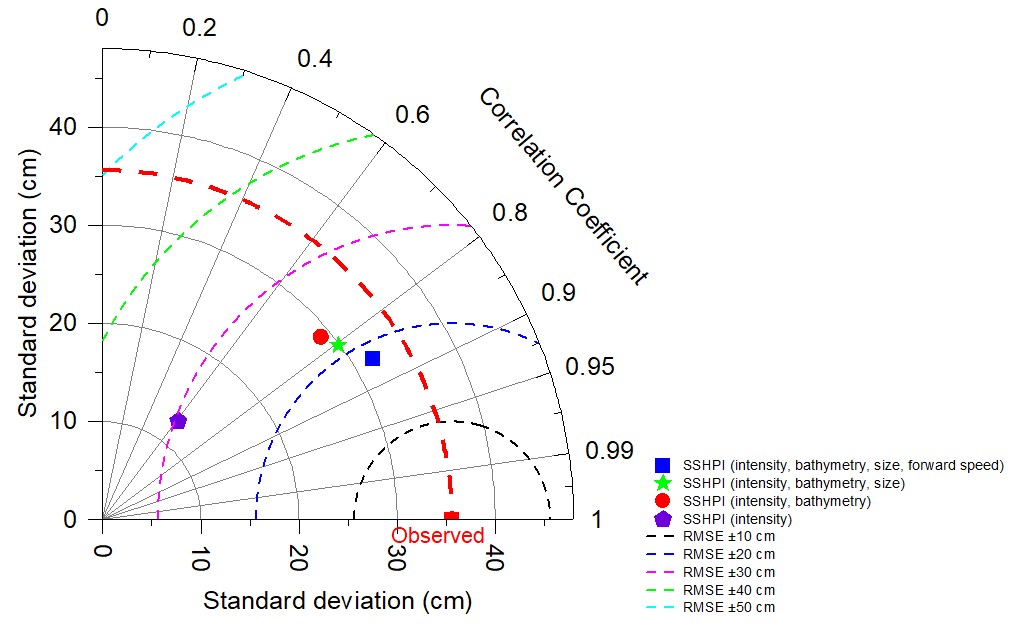


**Figure S3.** Taylor diagram describing the performance of SSHPI (for semi-enclosed bays; Japan) by comparing with the reduced versions of SSHPI. The azimuthal angle represents correlation, the radial distance the standard deviation, and the semicircles centered at the “Observed” marker the root mean square error. The red dashed line constitutes the standard deviation of observed peak storm surge heights in bays (n = 47).

**Hindcasting major storm surge events in Japan**

| TC no. | TC name | Observed tide station (type of coast) | Observed peak storm surge^52^ (cm) | Estimated surge (cm) (eq.4) | SSHPI (eq.3) | SSHPI (intensity) |
| --- | --- | --- | --- | --- | --- | --- |
| 199119 | Mireille | Oura (bay) | 217 | 197 | 5.45 | 3.61 |
| 199019 | Flo | Nagoya (bay) | 172 | 153 | 3.71 | 2.56 |
| 201821 | Jebi | Osaka (bay) | 161 | 111 | 2.01 | 2.89 |
| 199426 | Orchid | Nagoya (bay) | 153 | 167 | 4.26 | 2.56 |
| 201919 | Hagibis | Harumi (bay) | 138 | 172 | 4.47 | 2.56 |
| 201824 | Trami | Nagoya (bay) | 133 | 150 | 3.60 | 2.56 |
| 201721 | Lan | Maisaka (open) | 124 | 113 | 2.12 | 2.56 |
| 201721 | Lan | Mera (open) | 121 | 78 | 0.71 | 2.56 |
| 201721 | Lan | Harumi (bay) | 120 | 126 | 2.61 | 2.56 |
| 201115 | Roke | Harumi (bay) | 119 | 106 | 1.84 | 2.56 |
| 201721 | Lan | Akabane (open) | 117 | 113 | 2.12 | 2.56 |
| 201824 | Trami | Murotomisaki (open) | 115 | 73 | 0.50 | 2.56 |
| 201115 | Roke | Akabane (open) | 112 | 89 | 1.15 | 2.56 |
| 201115 | Roke | Maisaka (open) | 112 | 82 | 0.88 | 2.56 |
| 201821 | Jebi | Wakayama (bay) | 107 | 76 | 0.65 | 2.89 |
| 201915 | Faxai | Harumi (bay) | 101 | 103 | 1.71 | 2.56 |
| 201824 | Trami | Kushimoto (open) | 99 | 73 | 0.50 | 2.56 |
| 201919 | Hagibis | Mera (open) | 90 | 70 | 0.41 | 2.56 |
| 199918 | Bart | Kagoshima (bay) | 89 | 83 | 0.91 | 2.89 |
| 201824 | Trami | Toba (open) | 86 | 97 | 1.47 | 2.56 |
| 200416 | Chaba | Kagoshima (bay) | 85 | 88 | 1.11 | 2.56 |
| 201821 | Jebi | Murotomisaki (open) | 84 | 70 | 0.38 | 2.89 |
| 201721 | Lan | Omaezaki (open) | 84 | 78 | 0.71 | 2.56 |
| 201217 | Jelawat | Naha (open) | 80 | 70 | 0.38 | 3.24 |
| 199313 | Yancy | Aburatsu (open) | 77 | 69 | 0.34 | 3.24 |
| 200416 | Chaba | Aburatsu (open) | 74 | 65 | 0.21 | 2.56 |
| 199426 | Orchid | Toba (open) | 71 | 81 | 0.83 | 2.56 |
| 201824 | Trami | Wakayama (bay) | 70 | 80 | 0.78 | 2.56 |
| 201824 | Trami | Naha (open) | 67 | 67 | 0.26 | 2.89 |
| 199019 | Flo | Toba (open) | 66 | 79 | 0.77 | 2.56 |
| 201721 | Lan | Uchiura (bay) | 66 | 68 | 0.33 | 2.56 |
| 201115 | Roke | Uchiura (bay) | 63 | 68 | 0.31 | 2.56 |
| 200704 | Man-yi | Naha (open) | 62 | 71 | 0.42 | 3.61 |
| 200216 | Sinlaku | Naha (open) | 56 | 64 | 0.17 | 2.56 |
| 199918 | Bart | Makurazaki (open) | 54 | 68 | 0.32 | 2.89 |
| 199918 | Bart | Naha (open) | 51 | 66 | 0.22 | 3.24 |
| 200422 | Ma-on | Uchiura (bay) | 42 | 63 | 0.10 | 2.56 |

**Table S1.** TC intensity-based (*V_max_* ≥ 80-kt during landfall time frame; n = 37) definition of major storm surges

**Application Hurricane Katrina (2005)**

SSHPI values for Hurricane Katrina were calculated from NHC forecast advisory^71^ issued 24-h and 12-h before the storm made landfall. Forecast SSHPI (Fig. S4 (a)) indicates that the expected surge hazard was extremely high (> 7.5 m), particularly along the coasts surrounding the landfall location. The 12-h before landfall SSHPI forecast (Fig. S4 (b)) remained high (> 6.0 m) amid Katrina’s weakening winds. These forecast SSHPI surge values were compared with observed values (at Pass Christian, Dauphin Island AL, Pilots Station East S.W. Pass LA)^69,70^ and show reasonable agreement.

While this study has focused primarily on the single storm (i.e., Katrina) peak surge potential, the forecast map (Fig. S4) provides an example of how the spatial extent of surge hazard could be communicated using SSHPI. Storm surge heights are generally derived from point-scale observations (i.e., tide gauges), describing the distribution of extreme surge events at a specific location. But generally, the number of tide gauges are few and some places have no access to observed tide stations, implying a limited ability to describe distributions at larger spatial scales. Nonetheless, this limitation can potentially be overcome utilizing the SSHPI forecast as shown in Fig S4.


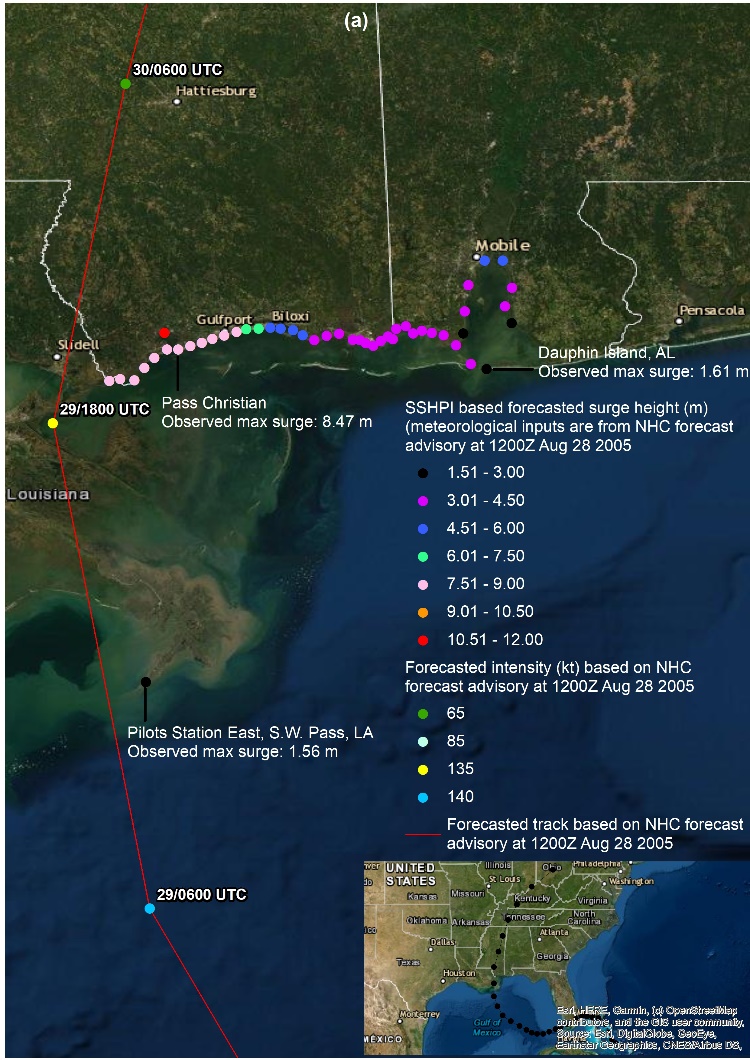

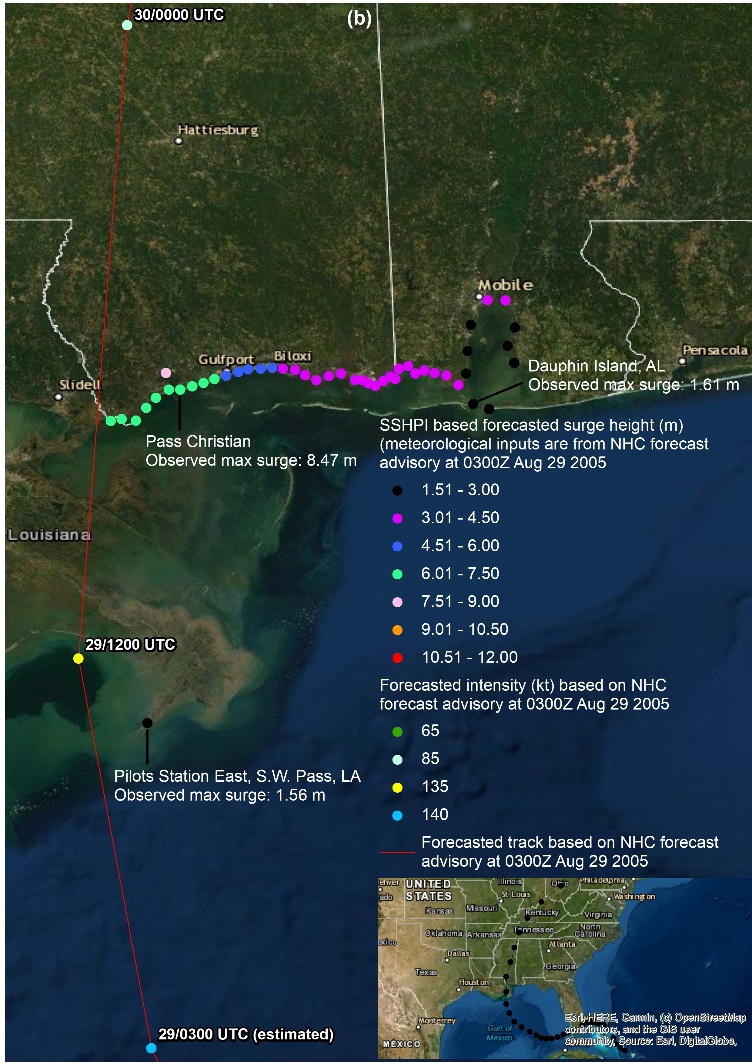


**Figure S4**. SSHPI forecasts for Hurricane Katrina (2005) based on NHC forecast advisory issued at (a) 1200Z Aug 28 2005 (approx. 24-h before landfall); (b) 0300Z Aug 29 2005 (approx. 12-h before landfall). SSHPI based forecasted points fell right side of the forecasted track and located within the vicinity of *R_50_*. Color of the surge height circle varies by the magnitude of the storm surge hazard potential. All maps are created using ArcMap (v. 10.2).
